# A novel method detecting the key clinic factors of portal vein system thrombosis of splenectomy & cardia devascularization patients for cirrhosis & portal hypertension

**DOI:** 10.1186/s12859-019-3233-3

**Published:** 2019-12-30

**Authors:** Mingzhao Wang, Linglong Ding, Meng Xu, Juanying Xie, Shengli Wu, Shengquan Xu, Yingmin Yao, Qingguang Liu

**Affiliations:** 1 School of Computer Science, Shaanxi Normal University, Xi’an, 710062 People’s Republic of China; 2 College of Life Sciences, Shaanxi Normal University, Xi’an, 710062 People’s Republic of China; 3 Department of Hepatobiliary Surgery, The First Affiliated Hospital of Xi’an Jiaotong University, Xi’an, 710061 People’s Republic of China; 4 Department of General Surgery, 513 Hospital of PLA, Lanzhou, 732750 People’s Republic of China

**Keywords:** Liver cirrhosis, Portal vein system thrombosis (PVST), Portal hypertension, Splenectomy, Cardia devascularization, Feature selection, SVM, Discernibility, Independence, Risk degree

## Abstract

**Background:**

Portal vein system thrombosis (PVST) is potentially fatal for patients if the diagnosis is not timely or the treatment is not proper. There hasn’t been any available technique to detect clinic risk factors to predict PVST after splenectomy in cirrhotic patients. The aim of this study is to detect the clinic risk factors of PVST for splenectomy and cardia devascularization patients for liver cirrhosis and portal hypertension, and build an efficient predictive model to PVST via the detected risk factors, by introducing the machine learning method. We collected 92 clinic indexes of splenectomy plus cardia devascularization patients for cirrhosis and portal hypertension, and proposed a novel algorithm named as RFA-PVST (Risk Factor Analysis for PVST) to detect clinic risk indexes of PVST, then built a SVM (support vector machine) predictive model via the detected risk factors. The accuracy, sensitivity, specificity, precision, F-measure, FPR (false positive rate), FNR (false negative rate), FDR (false discovery rate), AUC (area under ROC curve) and MCC (Matthews correlation coefficient) were adopted to value the predictive power of the detected risk factors. The proposed RFA-PVST algorithm was compared to mRMR, SVM-RFE, Relief, S-weight and LLEScore. The statistic test was done to verify the significance of our RFA-PVST.

**Results:**

Anticoagulant therapy and antiplatelet aggregation therapy are the top-2 risk clinic factors to PVST, followed by D-D (D dimer), CHOL (Cholesterol) and Ca (calcium). The SVM (support vector machine) model built on the clinic indexes including anticoagulant therapy, antiplatelet aggregation therapy, RBC (Red blood cell), D-D, CHOL, Ca, TT (thrombin time) and Weight factors has got pretty good predictive capability to PVST. It has got the highest PVST predictive accuracy of 0.89, and the best sensitivity, specificity, precision, F-measure, FNR, FPR, FDR and MCC of 1, 0.75, 0.85, 0.92, 0, 0.25, 0.15 and 0.8 respectively, and the comparable good AUC value of 0.84. The statistic test results demonstrate that there is a strong significant difference between our RFA-PVST and the compared algorithms, including mRMR, SVM-RFE, Relief, S-weight and LLEScore, that is to say, the risk indicators detected by our RFA-PVST are statistically significant.

**Conclusions:**

The proposed novel RFA-PVST algorithm can detect the clinic risk factors of PVST effectively and easily. Its most contribution is that it can display all the clinic factors in a 2-dimensional space with independence and discernibility as y-axis and x-axis, respectively. Those clinic indexes in top-right corner of the 2-dimensional space are detected automatically as risk indicators. The predictive SVM model is powerful with the detected clinic risk factors of PVST. Our study can help medical doctors to make proper treatments or early diagnoses to PVST patients. This study brings the new idea to the study of clinic treatment for other diseases as well.

## Background

Portal vein system thrombosis (PVST) refers to the blockage or narrowing of the portal vein, splenic and superior mesenteric veins, or intrahepatic portal vein branches, by a thrombus [1]. It is relatively rare and its clinical manifestations range from asymptomatic to severe complications including fever, abdominal pain, nausea, vomiting, and ileus [2]. The formation of PVST could increase the risk of upper gastrointestinal bleeding, hepatic coma or even fatal intestinal necrosis [3]. Moreover, PVST imposes difficulty on further liver transplantation [4, 5]. With the development of imageological examination, more and more studies have shown that the incidence of PVST after splenectomy is significantly higher than previously reported. The reported incidence of PVST after splenectomy is different greatly, ranging from 0.36% [6] to even 80% [7]. Why are there so much inconsistence in the incidence of post-splenectomy PVST? It comes from the difference in examination methods, types of study, time and frequency of postoperative examinations, and the underlying diseases, etc. [8]. Up to now, the specific mechanisms leading to the formation of PVST after splenectomy are not known. It is generally agreed that hemodynamic changes of the portal venous system [9–11], blood hypercoagulability [3], cecum induced by splenic vein ligation [12], local inflammatory reaction [13], and irrational use of coagulants [14] are all important factors affecting the occurrence of PVST. Some studies also demonstrated that the formation of PVST was related to the volume of spleen, diameter of portal vein, prothrombin time (PT), plasma D-dimer level, and the function and quality of platelet rather than the count of platelet [15–18]. So far, it has been controversial in the role of early prophylactic anticoagulation in preventing PVST. This is because of concerning the risk of inducing bleeding, especially in the cirrhotic patients [19–21]. However, in the last decade some studies demonstrated that both pro- and anticoagulation elements were concomitantly reduced in liver cirrhosis patients [22, 23], and the occurrence of bleeding for these patients was mainly due to the severity of portal pressure, endothelial dysfunction and bacterial infections, but not the disturbed hemostasis [24]. These studies provide the fundamental science for the prophylactic application of anticoagulation in these patients. Although the study to PVST has attracted many researchers [25–30] and some of them have found that prophylactic anticoagulation therapy can effectively prevent PVST after splenectomy even to cirrhotic patients [31], there are not any standard regimen for PSVT prophylaxis having been developed, and furthermore there are not any researchers focusing on detecting risk factors of PVST after splenectomy in cirrhotic patients by introducing machine learning to this field. Therefore we devote ourselves to this field.

We first propose a novel feature selection algorithm named RFA-PVST (Risk Factor Analysis for PVST) to detect the clinic risk factors of PVST, then we introduce the typical learning machine SVM to build the predictive model to PVST. We collect the clinic data of 92 splenectomy and cardia devascularization patients for cirrhosis and portal hypertension from the highest level hospital in PR China.

In our RFA-PVST, we propose the definition of discernibility and independence for each index to imply the capability of it in telling a PVST patient from non-PVST patients, and the differences of an index to other indices, respectively. The detected clinic indexes are with much higher discernibility and independence. The SVM model built on the detected risk factors can effectively tell PVST patients from non-PVST patients, and help medicine doctors to make proper cure decisions or early diagnoses to potential PVST patients. 5-fold cross validation experimental results on the aforementioned 92 clinic patients, and the statistic test between RFA-PVST and available famous feature selection algorithms demonstrate that the clinic risk factors detected by our RFA-PVST are statistically significant on which a very powerful predictive model is built.

## Results

This section will display the clinic risk factors of PVST detected by our proposed RFA-PVST, and the power of these risk factors in recognizing PVST patients by the performance of the SVM model based on them in terms of its accuracy shorted as Acc in the following of this paper, sensitivity, specificity, precision, F-measure, FPR (false positive rate), FNR (false negative rate), FDR (false discovery rate), AUC (area under ROC curve) and MCC (Matthews correlation coefficient). The performance comparison are shown between our RFA-PVST and the available feature selection algorithms including mRMR [32], SVM-RFE [33], Relief [34], S-weight [35] and LLEScore [36]. The statistic test results between our RFA-PVST and the aforementioned feature selection algorithms are also presented.

### Clinic risk factors of PVST

**Fig. 1:**
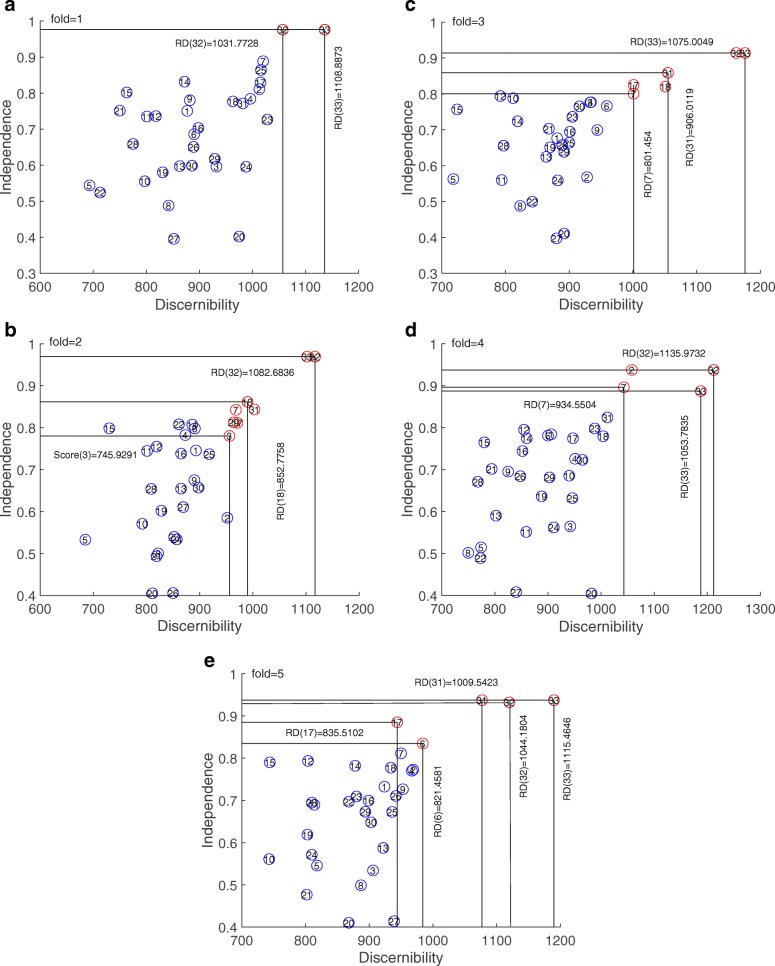
Scatter plots of clinic indexes of 5-fold cross validation experiments

Figure 1 displays the collection of all clinic indexes in circles in the 2-dimension space with discernibility as x-axis and independence as y-axis. The red circle indicates clinic risk factors, meaning the area of the rectangle enclosed by coordinate lines and axes is much bigger than the rest ones. Table 1 lists clinic indexes in descending order by their risk degrees in 5-fold cross validation experiments. The underlined bold font means the detected risk factors, corresponding to the red circle depicting clinic indexes in Fig. 1. Table 2 displays the performance of 5 different SVM models of 5-fold cross validation experiments on the test subsets in terms of Acc, AUC, sensitivity, specificity, precision, F-measure, FNR, FPR, FDR, and MCC. Table 3 displays the average results of 5-fold cross validation experiments in terms of same metrics as that in Table 2 under same conditions. The underlined bold fonts in Tables 2 and 3 mean the best results.

**Table 1:** the clinic indexes ranked in descending order in their risk degrees of 5-flod cross validation experiments

Folds	Clinic index *ID*s
1	**33**,**32**,7,25,17,2,4,31,18,23,14,9,1,16,15,6,12,11,24,26,29,21,3,30,13,28,19,10,8,20,5,22,27
2	**32**,**33**,**18**,**31**,**7,17,29,3**,14,6,22,4,25,1,16,12,9,11,30,15,13,2,27,28,19,23,24,10,8,21,5,26,20
3	**33**,**32**,**31**,**18**,**17**,**7**,6,4,3,30,23,9,10,12,16,21,25,1,14,26,29,19,15,13,2,28,24,11,22,5,8,20,27
4	**32,33,2,7,**31,23,18,17,1,6,30,4,12,14,10,16,29,25,15,26,9,19,21,3,28,24,11,13,5,20,22,8,27
5	**33,32,31,17,6**,7,2,4,18,9,14,1,26,12,25,16,23,22,29,15,30,28,11,13,19,3,24,5,8,10,27,21,20

The underlined bold fonts mean the detected risk factors

**Table 2:** Performance of PVST predictive models on different sets of risk indicators of 5-fold cross validation experiments

Fold (*C*, *γ*)	Acc	AUC	Sensitivity	Specificity	Precision	F-measure	FNR	FPR	FDR	MCC	# selected features
1 (2,0.0625)	0.68	**0.91**	0.64	**0.75**	0.78	0.70	0.36	**0.25**	0.22	0.38	1
	0.74	0.89	0.91	0.50	0.71	0.80	0.09	0.50	0.29	0.46	2
2 (0.25,0.25)	0.74	0.84	**1**	0.38	0.69	0.81	**0**	0.62	0.31	0.51	2
	**0.89**	0.84	**1**	**0.75**	**0.85**	**0.92**	**0**	**0.25**	**0.15**	**0.80**	8
3 (0.125,16)	0.61	0.85	0.70	0.50	0.64	0.67	0.30	0.50	0.36	0.20	2
	0.72	0.59	0.90	0.50	0.69	0.78	0.10	0.50	0.31	0.44	4
	0.61	0.76	0.80	0.38	0.62	0.70	0.20	0.62	0.38	0.19	6
4 (0.25,0.0625)	0.33	0.55	0.40	0.25	0.40	0.40	0.60	0.75	0.60	−0.35	1
	0.33	0.44	0.40	0.25	0.40	0.40	0.60	0.75	0.60	−0.35	2
	0.33	0.23	0.50	0.13	0.42	0.45	0.50	0.87	0.58	− 0.40	4
5 (0.5,0.125)	0.56	0.80	0.60	0.50	0.60	0.60	0.40	0.50	0.40	0.10	1
	0.56	0.70	0.50	0.63	0.63	0.56	0.50	0.37	0.37	0.13	3
	0.61	0.65	0.80	0.38	0.62	0.70	0.20	0.62	0.38	0.19	5
Average	0.59	0.70	0.70	0.45	0.62	0.65	0.30	0.55	0.38	0.18	–

The underlined bold fonts mean the best results

**Table 3:** Experimental results of algorithms of 5-fold cross validation experiments

Algorithms	Acc	AUC	Sensitivity	Specificity	Precision	F-measure	FNR	FPR	FDR	MCC
RFA-PVST	**0.59**	**0.70**	0.70	**0.45**	**0.62**	0.65	0.30	**0.55**	**0.38**	0.18
mRMR	0.53	0.53	0.78	0.20	0.55	0.64	0.22	0.80	0.45	NaN
SVM-RFE	0.56	0.44	**1.00**	0	0.56	**0.72**	**0**	1.00	0.44	NaN
Relief	0.56	0.56	**1.00**	0	0.56	**0.72**	**0**	1.00	0.44	NaN
S-weight	0.54	0.52	0.95	0.02	0.55	0.69	0.05	0.98	0.45	NaN
LLEScore	0.53	0.53	0.66	0.37	0.57	0.60	0.34	0.63	0.43	NaN

The underlined bold fonts mean the best results

### Statistic test results of RFA-PVST

Friedman’s test with *α* = 0.05 of our proposed RFA-PVST and mRMR, SVM-RFE, Relief, S-weight and LLEScore are displayed in Table 4 in terms of Acc, AUC, sensitivity, specificity, and precision of the SVM predictive models of PVST with the same number of risk indexes detected by each algorithm, respectively.

**Table 4:** The Friedman’s test results with *α* = 0.05 of our RFA-PVST and mRMR, SVM-RFE, Relief, S-weight and LLEScore

	Acc	AUC	Sensitivity	Specificity	Precision
*χ*^2^	11.7790	12.5166	37.4303	46.7109	17.0809
*df*	5	5	5	5	5
*p*	0.0379	0.0284	4.9e-07	6.507e-09	0.0043

The multiple comparison test between each pair of algorithms at the confidence level of 0.95 is displayed in Table 5 in terms of Acc, AUC, sensitivity, specificity, and precision. The upper triangle of each test shows the mean rank difference between algorithms, and the lower triangle the statistical significance between each pair of algorithms, where * is the tag of strong significance between corresponding algorithms in the corresponding metrics.

**Table 5:** Paired rank comparison of algorithms in Acc, AUC, sensitivity, specificity, and precision of predictive model built on clinic risk indicators to PVST detected by algorithms

*Acc*	RFA-PVST	mRMR	SVM-RFE	Relief	S-weight	LLEScore
RFA-PVST		1.7308	1.1923	1.1923	1.5769	1.0000
mRMR	*		−0.5385	−0.5385	− 0.1538	− 0.7308
SVM-RFE				0	0.3846	−0.1923
Relief					0.3846	−0.1923
S-weight						−0.5769
LLEScore						
***AUC***	RFA-PVST	mRMR	SVM-RFE	Relief	S-weight	LLEScore
RFA-PVST		1.7308	2.4231	1.7308	1.8077	1.7692
mRMR			0.6923	0	0.0769	0.0385
SVM-RFE	*			−0.6923	−0.6154	− 0.6538
Relief					0.0769	0.0385
S-weight						−0.0385
LLEScore						
**Sensitivity**	RFA-PVST	mRMR	SVM-RFE	Relief	S-weight	LLEScore
RFA-PVST		−0.5769	−2.2692	−2.2692	−1.8077	0.4615
mRMR			−1.6923	−1.6923	−1.2308	1.0385
SVM-RFE	*			0	0.4615	2.7308
Relief	*				0.4615	2.7308
S-weight	*					2.2692
LLEScore			*	*	*	
**Specificity**	RFA-PVST	mRMR	SVM-RFE	Relief	S-weight	LLEScore
RFA-PVST		1.3077	2.9615	2.9615	2.6923	0.2308
mRMR			1.6538	1.6538	1.3846	−1.0769
SVM-RFE	*			0	−0.2692	−2.7308
Relief	*				−0.2692	−2.7308
S-weight	*					−2.4615
LLEScore			*	*	*	
**Precision**	RFA-PVST	mRMR	SVM-RFE	Relief	S-weight	LLEScore
RFA-PVST		1.8462	1.8846	1.8846	2.3077	1.0769
mRMR	*		0.0385	0.0385	0.4615	−0.7692
SVM-RFE	*			0	0.4231	−0.8077
Relief	*				0.4231	−0.8077
S-weight	*					−1.2308
LLEScore						

## Discussion

This section will discuss all of the experimental results displayed in the section of results.

### Clinic risk factor discussion

The results in Fig. 1 disclose that our proposed metric *RD* is useful in detecting the clinic indexes with higher risk degree. The red circle clinic indexes in Fig. 1 comprise risk clinic indicators of PVST, and can be detected by our RFA-PVST automatically. The results in Fig. 1 reveal that the risk clinic factors for each fold experiment are variant for the variance of exemplars in each training subset of 5-fold cross validation experiments. However the number of risk factors of 5-fold cross validation experiments is from 2 to 8 with average 5. The common clinic indexes are anticoagulant therapy (with ID 32) and antiplatelet aggregation therapy (with ID 33) among 5 risk clinic indicator subsets detected by our proposed RFA-PVST. The clinic indexes of CHOL with ID 7, Ca with ID 17 and D-D with ID 31 appear 3 times among 5 subsets. This fact implies that anticoagulant therapy and antiplatelet aggregation therapy are the first two important risk indicators to predict PVST patients followed by the comparable important clinic indicators of CHOL, Ca and D-D.

The results in Table 1 disclose that antiplatelet aggregation therapy (with ID of 33) is the riskiest clinic index to PVST, followed by anticoagulant therapy (with ID of 32). The WBC (with ID of 20) and INR (with ID of 27) are the clinic indexes with the least risk degree causing PVST. In addition, the results in Table 1 tell us that although the training samples are variant, the first two clinic risk factors are same in each fold of 5-fold cross validation experiment, which further indicate that our proposed RFA-PVST algorithm is powerful in finding the risk clinic factor of PVST.

The results in Table 2 tell us that the performance of different PVST predictive models on test exemplars are variant in terms of Acc, AUC, sensitivity, specificity, precision, F-measure, FNR, FPR, FDR and MCC. The predictive model has got the highest AUC value of 0.91 with only one clinic index of whether antiplatelet aggregation therapy is treated or not, and the best specificity of 0.75 and the best FPR of 0.25 as well. The predictive model built on the 8 clinic indexes including anticoagulant therapy, antiplatelet aggregation therapy, RBC, D-D, CHOL, Ca, TT and weight, has got the highest PVST predictive accuracy of 0.89, and the best sensitivity, specificity, precision, F-measure, FNR, FPR, FDR and MCC of 1, 0.75, 0.85, 0.92, 0, 0.25, 0.15 and 0.8 respectively. Although its AUC is not the best one among 5-fold cross validation experiments, it has got the comparable good AUC value of 0.84. Therefore we can conclude that these 8 clinic indexes are important clinic indicators on which the sound prediction model can be built to predict whether PVST will take place or not for splenectomy with cardia devascularization patients for liver cirrhosis and portal hypertension.

The results in Table 3 tell us that our RFA-PVST can detect risk clinic indicators with which a SVM classifier can be built with best mean predictive accuracy, AUC, specificity, precision, FPR and FDR. Although this predictive model can only recognize 70% PVST patients in terms of sensitivity, not as good as that by SVM-RFE and Relief which can detect all PVST patients, our predictive model can detect 45% non-PVST patients while SVM-RFE and Relief cannot detect any one. This fact means that the predictive models by SVM-RFE and Relief exist the fatal error of recognizing all non-PVST patients as PVST ones, while the SVM classifier based on the risk indicators detected by our proposed RFA-PVST can make excellent tradeoff between sensitivity and specificity.

### Statistic test result discussion

It can be seen from the results in Table 4 that *p* < 0.05 holds for all metrics used to do statistic test, including Acc, AUC, Sensitivity, Specificity, and Precision. So we can conclude that the strong significant difference exist between our RFA-PVST and the compared algorithms, including mRMR, SVM-RFE, Relief, S-weight and LLEScore, that is the risk indicators detected by our RFA-PVST are statistically significant.

The multiple comparison test results in Table 5 in terms of accuracy (Acc), AUC, sensitivity, specificity, and precision of predictive models of PVST based on the risk indicators detected by the related algorithms reveal that our RFA-PVST can detect the risk clinic factors with much better predictive power to PVST for splenectomy plus cardia devascularization patients for liver cirrhosis and portal hypertension, compared to mRMR, SVM-RFE, Relief, S-weight and LLEScore. The results disclose the fact that our RFA-PVST is powerful in detecting the clinic risk indexes to predict whether PVST will happen or not on splenectomy plus cardia devascularization patients for liver cirrhosis and portal hypertension.

## Conclusions

A novel algorithm named RFA-PVST is proposed to detect the clinic risk indicators of PVST for splenectomy and cardia devascularization patients for liver cirrhosis and portal hypertension. The discernibility and independence are defined for each clinic index. All of the clinic indexes are scatted in a 2-dimensional space with independence and discernibility as y-axis and x-axis, respectively. Those clinic indexes in top-right corner of the 2-dimensional space are detected automatically as risk indicators. The SVM classifier is built on the detected risk indicators to predict whether the PVST will happen or not on a splenectomy plus cardiac devascularization patient for liver cirrhosis and portal hypertension.

5-flod cross validation experiments on the clinic data of 92 patients disclose that antiplatelet aggregation therapy is the riskiest clinic index, followed by anticoagulant therapy. Taking the two therapies may lead to PVST for splenectomy plus cardiac devascularization patients for liver cirrhosis and portal hypertension. CHOL, Ca, and D-D are also important risk factors. Anticoagulant therapy, antiplatelet aggregation therapy, RBC, D-D, CHOL, Ca, TT, and weight comprise the clinic risk indicators to PVST. The predictive model based on these 8 risk indicators is very powerful.

Furthermore, the comparison between our proposed RFA-PVST and available typical feature selection algorithms including mRMR, SVM-RFE, Relief, S-weight and LLEScore demonstrate that our RFA-PVST is very powerful to detect the risk clinic indicators to recognize PVST from non-PVST patients. The significant test between the aforementioned algorithms reveal that there is strong significant difference between our RFA-PVST and the famous available feature selection algorithms. In addition, it is fantastic that our study results are coincident with that from references [17, 37] about D-D is a clinic risk indicator of PVST.

We can conclude that our study is significant in the field of detecting risk factors causing PVST for splenectomy and cardia devascularization patients for liver cirrhosis and portal hypertension. It can help medical doctors to make proper treatments or early diagnoses to PVST patients. This study also provides a new idea to the clinic treatment of other diseases.

## Methods

This section will first introduce the data used in this paper, then the preprocessing method will be introduced for the data. It should be noted that we are authorized to use the data under the condition of deleting the privacy information of patients. Then the SVM learning machine will be briefly introduced. After that we will introduce the idea of our proposed novel algorithm RFA-PVST in detail, and the methods building a SVM classifier in the clinic risk factors detected by our RFA-PVST. Finally the statistical test method will be introduced to value the significant difference between our RFA-PVST and other classic methods.

### Data used in this paper

This subsection will cover the data information and the data preprocessing methods used in this paper.

### Raw data

We collected clinic data of 92 patients of splenectomy with cardia devascularization for liver cirrhosis and portal hypertension from one of the first level hospital in PR China. The patients are partitioned into two groups, one is composed of 52 patients with PVST, and the other is of 40 patients without PVST. The PVST group comprises 30 male and 22 female patients, and the ages of these patients are from 20 to 71 with average age and standard deviation of 47 ± 10. The non-PVST group is composed of 22 male and 18 female patients with ages from 27 to 77, and the average age with standard deviation is 47.9 ± 10.8. The descriptions of the data can be found in Table 6.

**Table 6:** Data information

	Male	Female	Age range (*μ* ± *σ*)
PVST	30	22	20~71 (47 ± 10)
non-PVST	22	18	27~77 (47.9 ± 10.8)

The causes of the cirrhosis and portal hypertension and the distributions for these 92 patients are here.

- 59 patients from HBV (Hepatitis B virus) cirrhosis, with 64.13% ratio.
- 8 patients of HCV (Hepatitis C Virus) cirrhosis, about 8.70% ratio.
- 7 patients for autoimmune cirrhosis with 7.61% ratio.
- 4 idiopathic cirrhosis patients with the ratio of 4.35%.
- 2 alcohol type cirrhosis patients with 2.17% ratio.
- 2 patients from idiopathic hypersplenism cirrhosis with the ratio of 2.17%.
- 1 splenic infarction cirrhosis patient with the ratio of 1.09%.
- 1 budd-chiari syndrome patient with the ratio of 1.09%.
- 1 gaucher disease patient with the ratio of 1.09%.
- 1 patient for both HBV + HCV with the ratio of 1.09%.
- 1 virus untyped cirrhosis patient with the ratio of 1.09%.
- 1 hypoferric anemia cirrhosis patient with the ratio of 1.09%.
- 1 patients for idiopathic thrombocytopenic purpura, and with 1.09%.
- 1 patient for primary hypersplenism with the ratio of 1.09%.
- 1 patient from portal cavernous transformation with 1.09% ratio.
- 1 patient for liver cirrhosis with 1.09%.

The clinic indexes of these 92 patients are listed in Table 7. There are 33 clinic indexes, including 6 countable clinic indicators such as age, gender, weight, bleeding volume, anticoagulant therapy, antiplatelet aggregation therapy, and the other 27 measurable indexes. The measuring clinic indexes are recorded daily or every other day after operations and the date was also recorded at the same time. There are two therapy for patients were adopted to prevent PVST after operations including anticoagulant therapy and antiplatelet aggregation therapy. The anticoagulant therapy comprises giving patients low molecular heparin calcium by hypodermic injection in 4100 IU/qd or 5000 IU/qd only, or combined with warfarin orally together. The antiplatelet aggregation therapy includes taking aspirins orally in 0.1~0.3 g/qd only, or together with dipyridamole in 25 mg/tid or 50 mg/tid.

**Table 7:** Clinic indexes of splenectomy with cardia devascularization for cirrhotic and portal hypertension patients

ID	Index name	ID	Index name	ID	Index name
1	Age	12	BUN (blood urea nitrogen)	23	NE1 (neutrophil count of 1st test)
2	Gender	13	CRE (creatinine)	24	NE2 (neutrophil count of 2nd test)
3	Weight	14	GLU (glucose)	25	PLT (Platelets)
4	BV (bleeding volume)	15	Na (Natrium)	26	PT (prothrombin time)
5	AST (aspartate aminotransferase)	16	K (Kalium)	27	INR (International normalized ratio)
6	ALT (alanine transaminase)	17	Ca (calcium)	28	APTT (activated partial thromboplastin time)
7	CHOL (cholesterol)	18	RBC (Red blood cell)	29	TT (thrombin time)
8	TBIL (total bilirubin)	19	HGB (hemoglobin)	30	FIB (fibrinogen)
9	DBIL (direct bilirubin)	20	WBC (White blood cell)	31	D-D (D dimer)
10	TP (total protein)	21	LY1 (lymphocyte count of 1st test)	32	Anticoagulant therapy,
11	ALB (albumin)	22	LY2 (lymphocyte count of 2nd test)	33	Antiplatelet aggregation therapy

### Data preprocessing

The age and the bleeding volume indexes use the original record value. Gender, anticoagulant therapy, and antiplatelet aggregation therapy are treated as Boolean variables, where male is 0 and female is 1, and without anticoagulant therapy is expressed as 0 and 1 otherwise, and without antiplatelet aggregation therapy is 0 and 1 otherwise. The median of measurable values is taken as the value for that measurable clinic indexes. If PVST occurred then the label for the patient is 1, which belongs to positive class, otherwise the label is − 1, belonging to negative class.

To avoid the influence on experimental results from variant measurement metrics for different clinic indexes, we successively normalize and discretize data in (1) and (2).

1 $$ {x}_{i,j}=\frac{x_{i,j}-\mathit{\min}\left({\mathbf{x}}_j\right)}{\mathit{\max}\left({\mathbf{x}}_j\right)-\mathit{\min}\left({\mathbf{x}}_j\right)} $$

where *x*_*i*, *j*_ is the specific value of the *j*^*th*^ index for the *i*^*th*^ patient, and *max*(**x**_*j*_), and *min* (**x**_*j*_) are the maximum and minimum value of the *j*^*th*^ index, respectively.

2 $$ {d}_{i,j}=\Big\{{\displaystyle \begin{array}{ll}-1& {x}_{i,j}<{\mu}_j-{\sigma}_j\\ {}\ 1& {x}_{i,j}>{\mu}_j+{\sigma}_j\\ {}\ 0& \kern1.5em else\end{array}} $$

where *μ*_*i*_ is the mean value of index *j* (1 ≤ *j* ≤ 33), and its standard deviation is *σ*_*i*_, then the discretized value for the index is *d*_*i*, *j*_ in (2).

### Support vector machines

SVM is a typical learning machine coined by Vapnik in 1920s [38]. It is based on the VC (Vapnik-Chervonenkis) dimension and the structure risk minimization with sound theoretic basics and concise mathematic model. It is a learning machine for small exemplars, and has got best generalization by making the optimal trade-off between the model complexity and the learning ability. SVM has been widely used in biomedical filed, and has greatly influenced the diagnosis and predictions of diseases [39–42]. The characteristic of SVM is that it maps the samples in low dimensional input space into high-dimensional feature space via kernel functions, so that the inseparable exemplars in low dimensional input space has become separable in high-dimensional feature space by an optimal hyperplane.

The popular used kernel functions are here.

- linear kernel functions: *K*(**x**, **x**') = **x** ⋅ **x**'.
- polynomial kernel function: *K*(**x**, **x**') = (**x** ⋅ **x** '  + 1)^*d*^, *d* is positive integers.
- radial basis kernel function: *K*(**x**, **x**') = exp(−‖**x** − **x**'‖^2^/*σ*^2^), *σ* is positive real.

### RFA-PVST algorithm

Feature selection is to detect several features from original ones to construct the feature subset making a specific criterion optimized [43]. The nature of feature selection is to display samples in a low dimensional space by those selected several features while preserving the pattern of samples as that in its original high dimensional space as much as possible [43]. It is usually implemented by erasing redundant and less important features while preserving the important ones. The selected features not only can preserve the classification power of original system, but also can reduce the complexity of classification model while improving its generalization [43–45]. The selected features preserve their physical properties with good interpretability, such that feature selection study has been paid much more attention by experts from statistics and machine learning fields, and has been widely applied to disease diagnoses [39–42]. The selected features do help medicine doctors to make proper decisions and take proper diagnoses to related patients.

We propose RFA-PVST algorithm to detect clinic risk factors of PVST for splenectomy and cardia devascularization patients for cirrhosis and portal hypertension, so as to build the predictive model for PVST via the detected risk factors. The 92 post-splenectomy and cardia devascularization patients comprise exemplars for liver cirrhosis and portal hypertension, and their clinic indexes as features. The detecting clinic risk indexes is in fact a feature selection procedure.

We define the discernibility and independence for each clinic index, and plot the curve of independence with discernibility for all clinic indexes in a 2-dimensional space with discernibility and independence as x-axis and y-axis, respectively. All clinic indexes in top-right corner of the 2-dimensional space comprise risk factors for they are with both comparatively high discernibility and high independence, while the less risk ones lie in bottom-left corner. To quantify how much contributions of a clinic index to telling a PVST patient form non-PVST patients, we define the risk degree for each clinic index as the product of its discernibility and its independence, that is, the area of the rectangle enclosed by coordinate lines and axes in the 2-dimensional space. Consequently the clinic indexes with much higher risk degree than the rest ones are detected out and the SVM classifier is built based on the risk factors to predict whether the splenectomy and cardia devascularization patients for liver cirrhosis and portal hypertension are PVST patients or not.

Let training dataset **D** = {**x**_1_, **x**_2_, ⋯, **x**_*n*_} ∈ **R**^*m* × *n*^, where *m* is the number of patients and *n* the number of clinic indexes. We define *dis*_*j*_, *ind*_*j*_, and *RD*_*j*_ to express the discernibility, independence, and risk degree for the clinic index *j*(1 ≤ *j* ≤ *n*), respectively in (3)–(7).

#### Definition 1

*Discernibility*: Let *N*_0_ and *N*_1_ be the number of patients with and without PVST, respectively, and *S*(*j*) be the statistics of Wilcoxon signed rank test for clinic index *j*, *x*_*i*, *j*_ is the value of sample *i* in its clinic index *j*, then the discernibility *dis*_*j*_ of clinic index *j* is defined in (3), and *S*(*j*) is calculated in (4).

3 $$ di{s}_j=\mathit{\max}\left\{{N}_0\ast {N}_1-S(j),\kern1em S(j)\right\} $$

4 $$ S(j)=\sum \limits_{k=1}^{N_0}\sum \limits_{i=1}^{N_1}\chi \left(\left({x}_{i,j}-{x}_{k,j}\right)\le 0\right) $$

where $$ \chi \left(\cdotp \right)=\Big\{{\displaystyle \begin{array}{l}1,\kern1em \left({x}_{i,j}-{x}_{k,j}\right)\le 0\\ {}0,\kern1em \mathrm{otherwise}\end{array}} $$.

From the **Definition** **1**, we can see that *dis*_*j*_ of clinic index *j* can express its discernibility between patients with PVST and without PVST very well, so it can be used to value whether the clinic index *j* is a risk factor or not of causing PVST for splenectomy and cardia devascularization patients for liver cirrhosis and portal hypertension.

#### Definition 2

*Independence*: The independence *ind*_*j*_ of clinic index *j* is defined in (5), where **x**_*j*_ and **x**_*k*_ are vectors of clinic index *j* and *k*. It is a negative exponential function of the correlation coefficient *pr* between clinic index *j* and its most correlated clinic index *k* with higher discernibility. For the clinic index *j* with the highest discernibility to PVST, its independence is defined as the negative exponential function of the correlation coefficient *pr* between *j* and its least correlated clinic index *k*. This correlation coefficient *pr* can be any kind of parameters to express the correlation between two variables. We adopt Pearson coefficient in our study. In order to unify the positive or negative correlation between clinic indexes, we adopt the absolute of Pearson coefficient expressed in (6), where **X,Y** are vectors of any two clinic indexes, and $\overline{\mathbf{X}}$ is the mean vector of **X**, $\overline{\mathbf{Y}}$ the mean vector of **Y**.

5 $$ in{d}_j=\Big\{{\displaystyle \begin{array}{l}{\max}_k\left(\exp \left(- pr\left({\mathbf{x}}_j,{\mathbf{x}}_k\right)\right)\right),\kern1.12em di{s}_j=\max \left\{ di{s}_i|i=1,\cdots, n\right\}\\ {}\underset{k: di{s}_k\succ di{s}_j}{\min}\left(\exp \left(- pr\left({\mathbf{x}}_j,{\mathbf{x}}_k\right)\right)\right),\kern1em otherwise\end{array}} $$

6 $$ pr\left(\mathbf{X},\mathbf{Y}\right)\overline{=}\frac{\mid {\left(\mathbf{X}-\overline{\mathbf{X}}\right)}^T\left(\mathbf{Y}-\overline{\mathbf{Y}}\right)\mid }{\sqrt{{\left\Vert \mathbf{X}-\overline{\mathbf{X}}\right\Vert}^2{\left\Vert \mathbf{Y}-\overline{\mathbf{Y}}\right\Vert}^2}} $$

The above independence definition disclose that the less correlation of a clinic index with other indexes, the stronger is its independence, and vice versa. This definition is coincident with the principles in nature. In addition, the definition in (5) guarantees that the clinic index with the highest discernibility for PVST definitely has got the independence as high as possible, which further guarantees that it will be definitely selected as risk factors of PVST.

#### Definition 3

*Risk Degree (RD)*: The risk degree of clinic index *j* is defined as the product of its discernibility and independence in (7), which is the area of the rectangle enclosed by its coordinate lines and axes, where the discernibility is the x-coordinate and independence the y-coordinate.

7 $$ R{D}_j= di{s}_j\times in{d}_j $$

The main steps of the proposed RFA-PVST are described as follows.

**Input:** Training dataset *D* ∈ *R*^*m* × *n*^, *m* is the number of patients, *n* is the number of clinic indexes, Y is the label vector indicating PVST patients or not.

**Output**: Set *S* of risk factors.**BEGIN** let *S* = ∅, F = {all clinic factors}; FOR *j* = 1 to *n* DO BEGIN calculate *dis*_*j*_ for clinic index *j* in eq. (3); calculate *ind*_*j*_ for clinic index *j* in eq. (5); calculate *RD*_*j*_ for clinic index *j* in equation in (8); END //of FOR Plot all clinic indexes in the 2-dimensional space with discernibility as x-axis and independence as y-axis; Select clinic indexes in top-right corner to comprise set *S* of risk factors;**END**

### Constructing predictive models

5-cross validation experiments are conducted, and SVM learning machines with RBF (Radial Basis Function) kernel functions are adopted. The proposed RFA-PVST is used to detect risk factors of PVST. The SVM classifier is constructed based on the detected risk factors. The performance of this SVM classifier is compared to that based on the indices by available feature selection algorithms to evaluate the power of RFA-PVST in detecting factors to recognize PVST patients.

### Selecting parameters for SVM

The kernel function and its parameters are very important for a SVM learning machine [46]. We take RBF kernel function and grid search technique to find the optimal penalty parameter C and kernel function parameter *γ* for SVM. The grid search technique is to first set the specific range for C and *γ*, respectively, then test each pair of (C, *γ*) on training subset by cross validation experiments to find the best pair of (C, *γ*). Finally, the pair (C, *γ*) with the highest cross validation accuracy is the best pair parameters to be selected.

### Building SVM model for predicting PVST

5-fold cross validation experiments are done on our collected clinic data of splenectomy plus cardia devascularization for liver cirrhosis and portal hypertension. The patients with PVST and without PVST are partitioned into 5 balanced parts respectively, so as to get 5 subsets of exemplars for 5-fold cross validation experiments. The RFA-PVST algorithm is conducted on training subset to get risk factors to construct set *S*. Then we construct the new training subset *TS*_*new*_ whose exemplars only embodying risk factors from set *S*. The best pair of parameters (*C*, *γ*) is found on *TS*_*new*_. Finally the SVM classifier is built based on the best pair of parameters (*C*, *γ*) and the new training subset *TS*_*new*_ to predict PVST.

### Evaluation methods

The power of our proposed RFA-PVST is evaluated in two aspects. First, it is evaluated by the performance of the SVM classifier built on the selected risk indexes by proposed RFA-PVST. Second, it is evaluated by the significant statistic test between the SVM classifiers built on the risk indexes by RFA-PVST and by other popular feature selection algorithms.

### Model evaluation

The performance of the SVM classifier is tested by exemplars in test subset in terms of predictive accuracy shorted as Acc, sensitivity, specificity, precision, F-measure, FPR (False positive rate), FNR (False negative rate), FDR (False discovery rate), AUC(Area under an ROC curve) and MCC(Matthews correlation coefficient). ROC is the acronym of receiver operating characteristic curve, which is a very famous metric to evaluate a model. AUC is the quantity value of ROC [47, 48]. These metrics are defined in eqs. (8)–(17) based on the confusion matrix in Table 8. The power of our RFA-PVST is compared to the available feature selection algorithms including mRMR [32], SVM-RFE [33], Relief [34], S-weight [35] and LLEScore [36].

**Table 8:** Confusion matrix

	Predictive Positive class (PVST patients)	Predictive Negative class (non-PVST patients)
True Positive class (PVST patients)	True positive (TP)	False negative (FN)
True Negative class (non-PVST patients)	False positive (FP)	True negative (TN)

8 $$ Acc=\frac{TP+ TN}{TP+ FP+ FN+ TN} $$

9 $$ sensitivity=\frac{TP}{TP+ FN} $$

10 $$ specificity=\frac{TN}{FP+ TN} $$

11 $$ precision=\frac{TP}{TP+ FP} $$

12 $$ F- measure=\frac{2 precision\ast sensitivity}{precision+ sensitivity}=\frac{2 TP}{2 TP+ FP+ FN} $$

13 $$ FPR=\frac{FP}{FP+ TN}=1- specificity $$

14 $$ FNR=\frac{FN}{TP+ FN}=1- sensitivity $$

15 $$ FDR=\frac{FP}{TP+ FP}=1- precision $$

16 $$ MCC=\frac{TP\ast TN- FP\ast FN}{\sqrt{\left( TP+ FN\right)\ast \left( FP+ TN\right)\ast \left( TP+ FP\right)\ast \left( FN+ TN\right)}} $$

17 $$ AUC=\frac{\sum \limits_{i=1}^n\left({r}_i\right)-\frac{n_0\times \left({n}_0+1\right)}{2}}{n_0\times {n}_1} $$

where in (17), *n*_0_ *and  n*_1_ are the number of patients in the test subset with and without PVST respectively, and are referred to as the number of exemplars respectively in positive and negative class, and *n* = *n*_0_ + *n*_1_ is the total number of patients in the test subset, and *r*_*i*_ is the rank of the *i*th patient in descending order of its probability to be a PVST patient. The minimum start rank is set to 1.

From the above metric definitions, we can see that sensitivity expresses the ratio of detecting PVST patients from the true PVST patients, while specificity indicates the ratio of recognizing non-PVST patients from patients without PVST, and precision implies the ratio of the true PVST patients among the recognized PVST patients by our SVM predictive model. F-measure is the harmonic mean of precision and sensitivity.

### Statistic test

The statistic test is undertaken between the SVM classifiers built on the risk indexes detected by our RFA-PVST and by the aforementioned very popular feature selection algorithms from [32–36] to verify whether or not our proposed RFA-PVST is statistically significant. That is, the statistic test results can disclose whether or not the risk indicators detected by our RFA-PVST are statistically significant to predict PVST. The Friedman’s test [49, 50] is adopted to discover the significant difference between algorithms for it is considered preferable for comparing algorithms over datasets without any normal distribution assumption. Once the significant difference is detected, the multiple comparison test will be adopted as a post hoc test to detect the significant difference between pairs of algorithms. We’ll do Friedman’s test with *α* = 0.05 of algorithms in terms of Acc, AUC, sensitivity, specificity, and precision of the SVM predictive models of PVST with same number of risk indexes detected by each algorithm, respectively.

## Data Availability

The datasets used and/or analyzed during the current study are available from the corresponding author on reasonable request.

## Abbreviations

ALB: albumin
ALT: alanine transaminase
APTT: activated partial thromboplastin time
AST: aspartate aminotransferase
AUC: area under ROC curve
BUN: blood urea nitrogen
BV: bleeding volume
Ca: calcium
CHOL: cholesterol
CRE: creatinine
DBIL: direct bilirubin
D-D: D dimer
FDR: false discovery rate
FIB: fibrinogen
FN: false negative
FNR: false negative rate
FP: false positive
FPR: false positive rate
GLU: glucose
HBV: Hepatitis B virus
HCV: Hepatitis C Virus
HGB: hemoglobin
INR: International normalized ratio
K: Kalium
LLEScore: Locally Linear Embedding score
LY1: lymphocyte count of 1st test
LY2: lymphocyte count of 2nd test
MCC: matthews correlation coefficient
mRMR: minimum redundancy maximum relevance
Na: Natrium
NE1: neutrophil count of 1st test
NE2: neutrophil count of 2nd test
PLT: Platelets
PT: prothrombin time
PVST: portal vein system thrombosis
RBC: Red blood cell
RBF: radial basis function
RD: risk degree
RFA-PVST: risk factor analysis for PVST
ROC: receiver operating characteristic curve
SVM: support vector machine
SVM-RFE: SVM recursive feature elimination
TBIL: total bilirubin
TN: true negative
TP: total protein
TP: true positive
TT: thrombin time
VC: Vapnik-Chervonenkis
WBC: White blood cell
